# Complete mitochondrial genome of a tropical sea cucumber, *Stichopus chloronotus*

**DOI:** 10.1080/23802359.2021.1967218

**Published:** 2021-08-31

**Authors:** Xiaoying Chen, Yuping Sun, Hongxia Zhao, Junru Hu, Bing Chen, Huo Li, Wen Huang

**Affiliations:** a Laboratory of Aquatic Sciences, Key Laboratory of Animal Nutrition and Feed Science in South China, Ministry of Agriculture in Rural Affairs, Guangdong Key Laboratory of Animal Breeding and Nutrition, Institute of Animal Science, Guangdong Academy of Agricultural Sciences, Guangzhou, China; b Guangdong Provincial Engineering and Technology Research Center, Doctoral Workstation of Guangdong Province, Guangdong Jinyang Biotechnology Co. Ltd, Maoming, China

**Keywords:** Mitochondrial genome, *Stichopus chloronotus*, phylogenetic analysis

## Abstract

In this study, we report the complete mitochondrial genome of *Stichopus chloronotus.* The mitogenome was 16,247 base pairs (58.55% A + T content) in length, comprising a total of 37 genes, including 13 protein-coding genes, 22 transfer RNA genes and 2 ribosomal RNA genes. To resolve the phylogenetic position of *S. chloronotus*, we analyzed all mitochondrial protein-coding genes from 27 species within the Echinodermata. The results showed that *S. chloronotus* belonged to the family *Stichopodidae* and was more closely related to tropical *Stichopus* species (*S. horrens* and *S. monotuberculatus*) than to other species. Our results will be useful for evolutionary analysis of sea cucumber species.

Sea cucumbers are an important group of benthic organisms comprising abundant and diverse ecologically and economically important species (Liao 1997). These organisms are widely distributed in oceans worldwide from depths of ten thousand meters in the sea or trenches to intertidal zones. They mainly live-in reefs, sediment and seaweed and are often overgrown (Gallo et al. 2015; Mu et al. 2018; Yang et al. 2019). *Stichopus chloronotus* (*S. chloronotus*) is widely distributed in the Indo-Western Pacific region, and mainly inhabits in the Xisha Islands of Hainan Province in China (Liao 1997). *S. chloronotus* contains sticholoroside, stichostatin I, fucosylated chondroitin sulfate (Mou et al. 2020), fatty acids (Fredalina et al. 1999) and other compounds. The active ingredients of *S. chloronotus* were reported to expedite the wounds healing (Mohamed et al. 2015). Complete mitochondrial sequencing is an important method for analyzing phylogenetic relationships and identifying species of sea cucumbers (Rowe and Richmond 2004; Cheng et al. 2019, 2020). However, the mitochondrial genome of *S. chloronotus* has not yet been revealed. In this study, we constructed a phylogenetic tree of 27 species in the sea cucumber family. These molecular data will be useful for the evolutionary analysis of tropical sea cucumbers.

The specimen was obtained from Sanya City, Hainan Province, China (N18°2021′, E109°4708′). Total DNA was extracted from longitudinal muscle tissue by using a TIANamp Marine Animals DNA Kit (Tiangen Biochemistry Technology Co., Ltd., China) and deposited at Guangdong Academy of Agricultural Sciences in Guangzhou, China (http://www.gdias.net/, Xiaoying Chen is the contact person xiaoyingchen05@126.com) under the voucher number GDAAS-IAS-AQUA-CA-202008102. The DNA was sequenced on an Illumina HiSeq 4000 sequencing platform (150-bp paired-end reads were generated) at Shenzhen Huitong Biotechnology Co., Ltd., China (Li et al. 2021). Raw sequence reads were edited using the NGS QC Tool Kit v2.3.3 and assembled into contigs using the *de novo* assembler SPAdes 3.11.0 (Dmitry et al. 2016), and the complete mitogenome was deposited on the NCBI website (https://www.ncbi.nlm.nih.gov/genbank/) with the accession number MW218897.1. The protein-coding genes (PCGs; tRNA and rRNA genes) were annotated by using MITOS Web Server BETA (http://bloodymary.bioinf.uni-leipzig.de/mitos/index.py). The tRNA genes were identified by using RNAscan-SE software (Lowe and Chan 2016). The phylogenetic relationships were reconstructed with the PCGs by means of maximum-likelihood (ML) (GTR + G + I model) analysis by using MEGA-X software with 1000 replicates (Kumar et al. 2018; Li et al. 2021).

The complete mitogenome of *S. chloronotus* was 16,247 bp in length (GenBank accession number: MW218897.1) and contained the typical set of 13 PCGs, 22 tRNA genes, 2 rRNA genes, and one putative control region. The overall base composition of the mitogenome was estimated to be 31.48% A, 27.07% T, 25.39% C and 16.06% G, with a high A + T content of 58.55%. In the phylogenetic tree, *S. chloronotus* was distinct from the other species, forming an independent branch (Figure 1). The results showed that *S. chloronotus* belonged to the family Stichopodidae, and we revealed for the first time that *S. chloronotus* was more closely related to the tropical *Stichopus* species *S. horrens* and *S. monotuberculatus* than to other echinoderm species.

**Figure 1.**
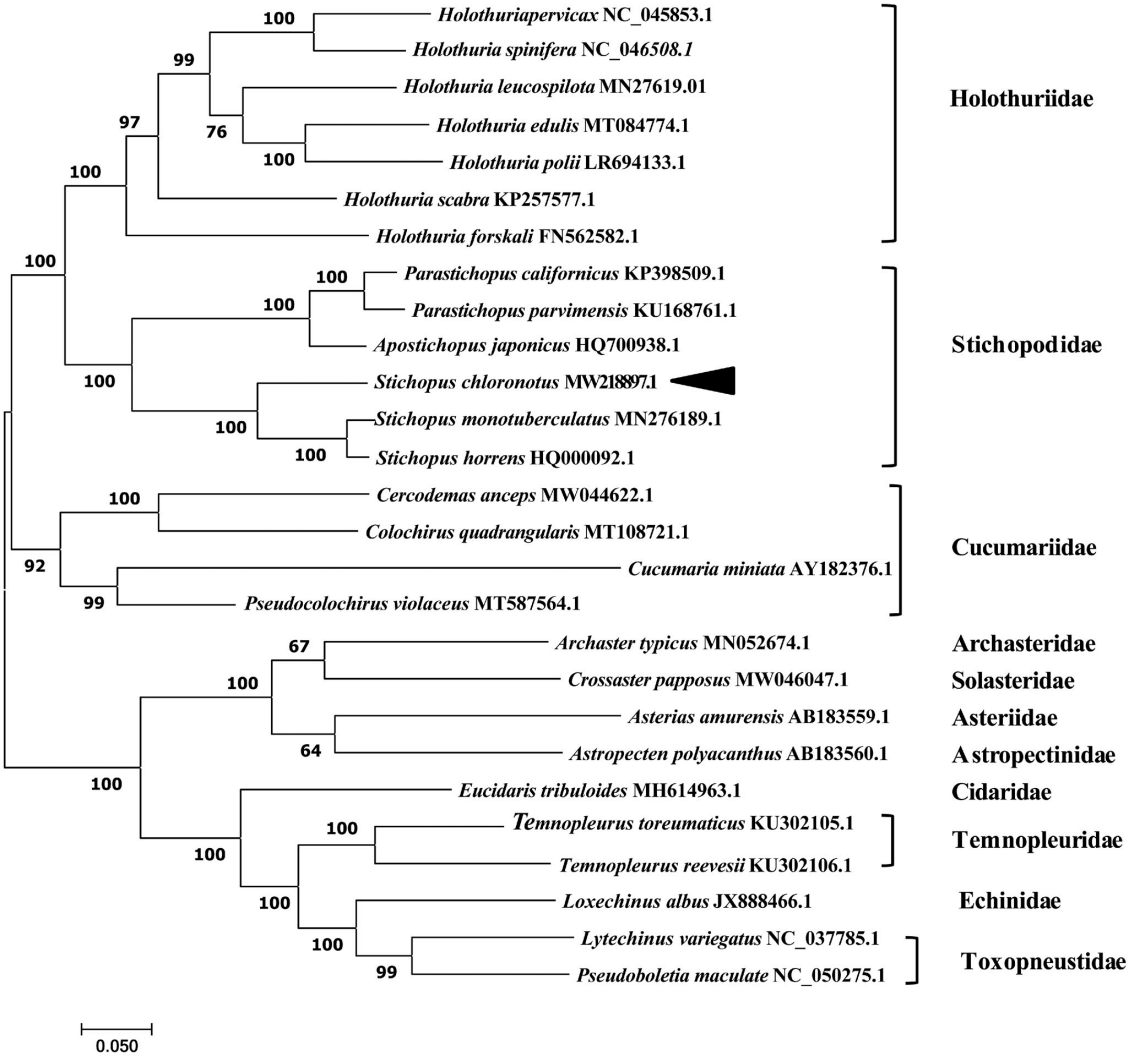
Maximum-likelihood (ML) phylogenetic tree based on the complete mitochondrial genomes of 27 species. Values along branches correspond to ML bootstrap percentages. The phylogenetic position of *Stichopus chloronotus* was marked with a dark-triangle.

## Data Availability

The genome sequence data that support the findings of this study are openly available in GenBank of the NCBI at (https://www.ncbi.nlm.nih.gov/) under accession no. MW218897.1. The associated BioProject, SRA, and Bio-Sample numbers are PRJNA704715, SRR13787362and SAMN18043597, respectively.
